# Anatomical variations of the thyroid gland: An experimental cadaveric study

**DOI:** 10.1016/j.amsu.2021.102823

**Published:** 2021-09-08

**Authors:** Ali Al-Azzawi, Tokiharu Takahashi

**Affiliations:** a The University of Manchester, Faculty of Biology, Medicine and Health, Manchester, UK; b University of Manchester School of Medicine: The University of Manchester Faculty of Biology Medicine and Health, UK

**Keywords:** Thyroid gland, Goitre, Anatomy, Variations, Dissection, Pyramidal lobes, Vasculature, STA

## Abstract

**Introduction:**

The thyroid gland displays numerous variations in its anatomy. Understanding the variations that occur can benefit diagnosis of thyroid disorders and improve management. The aim of this study was to investigate how factors such as age and sex may influence variations in the thyroid.

**Methods:**

Twenty cadavers (10 males & 10 females) with a mean age of 78 were dissected. Variations in anatomy and vasculature were examined. Correlation between age and thyroid size was tested for significance using GraphPad prism 7.

**Results:**

Most cadavers, 65%, had the superior thyroid artery originating from the external carotid artery, while 25% were from the bifurcation and 10% from the common carotid. The average weight for thyroids was 19.9 g in males and 13.9 g in females. A significant negative correlation was found between age and thyroid size.

**Discussion:**

Thyroid gland variations, such as pyramidal lobes which affected 30% of cadavers, could impact medical interventions. Evidence from this study has confirmed the high incidence of such variations emphasising the requirement for preoperative imaging.

## Highlights

• Variations in the thyroid gland anatomy can cause complications during thyroidectomy due to discrepancies in venous drainage and origin of the Superior thyroid artery
• The origin of the STA can vary between the common carotid, bifurcation or the external carotid. The origin on the external carotid can vary as it can be seen to arise higher up on the external carotid which leads to confusion with the lingual artery
• Pyramidal lobes are present in a significant number of cadavers. As these appear to be functional, they can be clinically useful in cases of hyperthyroidism due to overactivity of the thyroid gland
• Standardisation on the dissection process can allow further research on a bigger sample to improve reliability of the study. This can allow more accurate estimation of the variations seen.

## 1 Introduction

The thyroid gland is richly vascularised by branches of the external carotid artery and the subclavian artery. These arteries give rise to the Superior Thyroid Artery (STA) and Inferior Thyroid Arteries (ITA) respectively. The vessels from each side anastomose with the vessels from the contralateral side over the anterior surface of the gland [1]. Three thyroid veins drain the gland; the superior and middle thyroid veins both drain into the internal jugular vein, whilst the inferior thyroid veins drain into the brachiocephalic vein [2]. Although the STA which supplies the thyroid gland is known to be the first branch of the external carotid artery, the origin of which has been reported to vary significantly in different individuals. The STA has been shown to arise from the Common Carotid, External Carotid Artery (ECA) and sometimes directly from the bifurcation of the common carotid [3]. Knowledge of the prevalence of these variations can be vital for surgery to appropriately ligate the STA and not mistake it with the lingual artery which is the second branch of the ECA [4].

The recurrent laryngeal branch provides the parasympathetic nervous system innervation for the thyroid gland. The recurrent laryngeal nerve also supplies intrinsic muscles of the larynx to control the opening and closure of the vocal cords [5]. A close and variable relationship exists between this nerve and the inferior thyroid artery [6]. The nerve can be deep to the ITA branches, superficial to the ITA or travel in-between the ITA branches; superficial to one branch and deep to another [7]. Inferior thyroid vein drainage into the brachiocephalic can also be variable. For example, in most cases the right inferior thyroid vein drains into the right brachiocephalic, while the left inferior thyroid vein drains into the left brachiocephalic. In some cases, the right and left inferior thyroid veins form a common trunk to drain into the left brachiocephalic vein, this is known as a thyroid ima vein [8,9].

### 1.1 Lingual thyroid causing dysphagia and dyspnoea. Case reports and review of the literature

Some individuals display accessory thyroid tissue called the pyramidal lobe which can form as a result of a remnant of the thyroglossal duct. The shape, size and incidence of this varies greatly between different literatures [10]. The levator glandulae thyroideae muscle is present in some individual to connect the pyramidal lobe to the hyoid bone or thyroid cartilage [11]. The pyramidal lobe is of clinical significance as it can be affected by the same thyroid disorders as the rest of the gland [12]. In addition, the pyramidal lobe can absorb some of the radioactive iodine given as treatment of hyperthyroidism, hence, limiting the effectiveness of this treatment. Therefore, it is useful to be able to detect the presence of the pyramidal lobe and study its incidence within a certain population [13]*.*

This study investigated the thyroid glands in 20 cadavers to explore the incidence of anatomical variations present. Specifically, variations in the origin of the STA, drainage of the thyroid veins and the prevalence of a thyroid pyramidal lobe were investigated. This is useful clinically as accidental injury to any of the vasculature or surrounding structures can be minimised simply by being aware of any of these variations.

## 2 Materials and methods

### 2.1 Specimens

In this investigation, 20 embalmed cadavers (10 males and 10 females) were used from the Medical school dissection room. Age at death for the male cadavers ranged from 61 to 91 years old, with the mean age being 71. Age at death for the female cadavers ranged from 70 to 99 years old with a mean of 84 years. Death certificates were examined for all cadavers to ensure no endocrine disorders were directly the cause of death. All dissections were held in accordance with the Human Tissue Act (2004). Cadavers were embalmed by injecting embalming fluid into the carotid artery to fix the cadavers.

### 2.2 Dissection

Once the thyroid gland was identified, the surrounding structures and vessels were carefully dissected to display the complete anatomy of the region. The common carotid artery was separated from the vagus nerve and the internal jugular vein on both sides of the neck, these structures were traced to the level of the submandibular gland to note the branching patterns. The bifurcation of the common carotid artery was divided to clearly display the origin of the superior thyroid artery and level at which it branched from. The full branching pattern of the superior thyroid artery, as well as its relationship with the superior laryngeal branch of the vagus nerve was observed. The drainage of the thyroid gland was also investigated between cadavers, in particular the route taken by inferior thyroid veins.

### 2.3 Data collection

A digital calliper was used to measure the length, width and thickness of each lobe of the thyroid gland. The isthmus was assumed to be at the region between the 2nd and 3rd tracheal cartilage. The isthmus was also the region where the inferior thyroid vein descended as it drained the right and left lobes. Predetermined landmarks were set to ensure the process was standardised:

1. *Length*: Measured from the superior pole to the inferior pole of each lobe
2. *Width*: Measured along the widest point of the lobe from the isthmus to the lateral border
3. *Thickness*: Measured at the thickest point of the lobe at the midpoint from the superior to the inferior pole

### 2.4 Statistical tests

Numerical data were entered into GraphPad Prism 7 programme for statistical analysis. Firstly, Shapiro-Wilko test was used for normality testing in the data of interest to select for the most appropriate statistical test. All the data passed normality testing, apart from the overall thyroid weights. Therefore, a Man- Whitney *U* test was used to compare differences in weight (g) between males and females. Two-way ANOVA tests were used to determine if the right lobes were significantly larger, P < 0.05, than the left lobes in both sexes. Correlation coefficient analysis were conducted to look for a relationship between age and the size of the thyroid gland.

## 3 Results

### 3.1 Gross anatomy

Along the anterior of the thyroid the anastomosis of the superior thyroid artery could be seen, while the inferior thyroid artery was visualised posteriorly and lateral on the lobe. The inferior thyroid vein was observed travelling through the midline with branches from the right and left lobes. Meanwhile, the vagus nerve was found in the carotid sheath in between the common carotid artery and the internal jugular vein, this nerve gave off the recurrent laryngeal branch which supplies the gland.

A pyramidal lobe was observed in 6 out of the 20 cadavers (30% of males & 30% of females). All the pyramidal lobes in males appeared on the left side, whereas one of the 3 from a female cadaver arose from the right lobe. Nevertheless, in all cases the accessory lobe travelled superiorly to the hyoid bone where it attached via the levator glandulae thyroideae muscle. In addition, the pyramidal lobe clearly received supplementary blood supply from the STA on the respective side.

The average weight for the thyroid glands obtained from male cadavers was 19.9 g ± 15 while the thyroids of females was 13.9 g ± 10.3. The average dimensions of the thyroid gland lobes are outlines in Table 1.

**Table 1:** **Average dimensions***: The average dimensions of the right and left lobes from both sexes, measured by a digital calliper in millimetres (mm), along with the standard deviation values*.

	MALES			FEMALES		
	LENGTH (mm)	THICKNESS (mm)	WIDTH (mm)	LENGTH (mm)	THICKNESS (mm)	WIDTH (mm)
RIGHT	53.6 ± 9.1	13.7 ± 4.0	38.1 ± 3.4	44.8 ± 8.0	12.1 ± 4.0	28.2 ± 5.2
LEFT	48.8 ± 9.1	11.7 ± 3.3	31.8 ± 5.0	42.9 ± 7.6	11.7 ± 4.2	25.7 ± 4.5

### 3.2 Vasculature

The common carotid artery was observed to bifurcate at the level of the upper border of the thyroid cartilage. The superior thyroid artery originated from the bifurcation directly in 25% of the cadavers forming a trifurcation. In 10% of the specimens, the STA originated from the common carotid artery. The majority of the STA (65%) arose from the external carotid artery immediately superior to the bifurcation. The origin points did not vary between the right and left sides. Two unique dissections were recorded, one displayed the lingual artery arising from the bifurcation rather than the external carotid artery. In a second dissection the STA was seen to arise higher up in the external carotid artery, immediately inferior to the lingual artery.

The superior thyroid artery had four branches, the most superior of which was the hyoid branch which travelled along the hyoid bone. A lateral branch moved inferiorly to supply the sternocleidomastoid muscle, a cricothyroid branch supplied the larynx, while the main branch of interest was the superior laryngeal artery which supplied the thyroid gland. This artery was accompanied by the superior laryngeal nerve with close proximity. The superior laryngeal nerve was typically seen to divide below the hyoid bone into internal and external branches.

The inferior thyroid artery supplied the posterior and lateral aspects of the gland. ITA had a close association with the recurrent laryngeal nerve. The most common trend seen on the right side (50%) was the recurrent laryngeal nerve passing deep below the ITA, compared to only 30% on the left side. 25% of dissections demonstrated the nerve superior to one branch but inferior to another, therefore it was classed as going in-between the ITA. Most cadavers (80%) showed left to right asymmetry in this relationship. In other words, the association between the ITA and the recurrent laryngeal nerve on the right sides rarely matched what was seen on the left side.

In all cadavers, the superior and middle thyroid veins drained into the right and left internal jugular vein, however the drainage of the inferior thyroid vein differed. The most common trend in the male cadavers (60%) showed two separate inferior thyroid veins draining the right and left lobes into the brachiocephalic vein. In the remaining 40%, a common trunk (thyroid ima) was observed draining the right and left thyroid lobes into the left brachiocephalic vein. By contrast, the most common trend seen in the female cadavers (60%) showed the common trunk draining into the left brachiocephalic vein. Whereas only 40% had two inferior thyroid veins drain separately into the brachiocephalic.

## 4 Discussion

### 4.1 Relevance of variations in vasculature

Awareness of the possible variations in the route taken by an artery and nerve can be vital for a surgeon to be mindful of to avoid unnecessary damage. In the case of a thyroid gland surgery, this means being conscious of possible discrepancies in the origin of the superior thyroid artery and the proximity to the superior laryngeal nerve. As a result, the STA is usually ligated at the tip near the point of origin to avoid superior laryngeal nerve palsy[14,15].

In the 20 cadavers studied, it was found that the most common point of origin for the STA was from the ECA in both sexes, as seen in 65% of cadavers. While the least common origin (10%) was from the common carotid artery. These results matched findings by other researchers that found the STA originating from the ECA, bifurcation and common carotid artery in 71.5%, 21.5% and 7% respectively [3]. In contrast, some studies [16,17] proposed that the most common origin for the STA was from the bifurcation directly. One of the possible causes for this inconsistency can be due to the very short distance between where the STA would originate on the ECA and the bifurcation, making it difficult to distinguish the 2 sites accurately [18].

Variations in the relationship between the ITA and the recurrent laryngeal nerve also pose surgical relevance. During a thyroidectomy, the recurrent laryngeal nerve must be isolated to prevent it from being damaged when the ITA is ligated. Hence, a common risk of such surgery occurs due to the accidental damage of this nerve [19]. The nerve is also at risk of injury during surgeries involving the parathyroid gland and tracheostomy [21]. Previous research established that the relationship is highly variable and usually asymmetrical in humans [7]. This study confirms the asymmetry as only 20% of the cadavers showed similar anatomy between the right and left sides. One suggestion for the possible cause of asymmetry is due to the fact that the recurrent laryngeal nerve takes different routes on either side. On the right side, the nerve loops under the subclavian artery, while on the left side the nerve loops under the aortic arch before is ascends towards the thyroid gland [22]. Simply being aware of how the recurrent laryngeal nerve relates to the ITA has been reported to minimise complication associated with a thyroidectomy by up to 6% [20].

The most common type of drainage for the thyroid gland differed between males and females in this study. Males showed 2 separate drains from right and left lobes as the most common trend, while females were more likely to have thyroid ima veins. Other researchers have reported a range of data highlighting how variable this can be, such as having 3 inferior thyroid veins. From this, it can be concluded that having a common trunk or 2 separate right and left veins are the most common manifestations which occur at relatively the same incidence within a sample [8].

### 4.2 Pyramidal lobe

The prevalence of pyramidal lobes has previously been reported in up to 65.7% of a sample [23]. This study found no difference in the prevalence of pyramidal lobes between sexes despite previous research indicating higher incidence in males [24]. However, the incidence rate in the 20 cadavers studied varied greatly compared the previous research. This could simply be due to the difference in sample size, and a larger sample could be more reflective to that of previous research. Regardless, both studies suggest that pyramidal lobes are common within population. The persistence of the lobe following a thyroidectomy reduces the effectiveness of a treatment, as it can be responsible for excess thyroid hormone secretion and increased recurrence of disease. Furthermore, any remnant of a pyramidal lobe can absorb radioactive iodine hindering the treatment of thyroid tumours [25]. As a result, this study proposes thorough inspection of this region when a pyramidal lobe is suspected to ensure complete excision during a thyroidectomy. It is also proposes the use of serum thyroglobulin assay to confirm the success of a thyroidectomy in cases where a pyramidal lobe is suspected [26].

### 4.3 Gender differences

The size of thyroid glands from male cadavers were observed to be larger than thyroids obtained from female cadavers, despite only length and width being significantly different. The size difference is most likely to be dependent on differences in body weight and the fact that all the female cadavers were post-menopausal. Therefore, hormonal changes may have influenced the thyroid glands to shrink in females as post-menopausal women produce higher levels of Thyroid Stimulating Hormone (TSH) [27,28]. Hormone replacement therapy also alters hormone levels following menopause [29]. These factors could contribute to the higher incidence of shrinkage and thyroid disease predicted for older women [30,31].

### 4.4 Limitations

Variations investigated in this study were limited to cadavers from the University of Manchester dissecting room. Cadavers from other regions should also be investigated for these variations before more generalised conclusions can be obtained. The sample of 20 cadavers were also investigated during the same time period. An improvement of the research can involve a bigger sample investigated on separate occasions. A more standardised dissection procedure can also be beneficial to limit discrepancies in the observations.

Our results showed thyroid hormone independent positive associations between serum TSH and lipids, which were substantially influenced by gender and age. Males demonstrated more protective effects of low TSH against hyperlipidemia, while females showed more detrimental effects of high TSH on hyperlipidemia.

## 5 Conclusion

In conclusion, the study drew together multiple thyroid gland variations which could have an impact on treatment outcomes or diagnosis. This is particularly important due to variations in the origin of the STA. The prevalence of a pyramidal lobe was also found to be common in the population. Variations in the thyroid gland due to age and sex should highlighted as these were found to have a role in the variations observed. Due to the highly-varied anatomy of the region, the evidence supports the need for preoperative imaging and through inspection of the vasculature to minimise surgical complications.

## Provenance and peer review

Not commissioned, externally peer-reviewed.

## Ethical approval

None.

## Sources of funding

University of Manchester.

## Author contribution

Ali Al-Azzawi: Data collection, Analysis and Writing. Dr. Tokiharu Takahashi: Project supervisor. Stefan Gabriel: Project supervisor.

## Registry

1. Name of the registry:
2. Unique Identifying number or registration ID:
3. Hyperlink to your specific registration (must be publicly accessible and will be checked):

## Guarantor

Ali Al-Azzawi.

## Declaration of competing interest

There are no conflicts of interest.
